# Associations of Disease-Modifying Therapies With COVID-19 Severity in Multiple Sclerosis

**DOI:** 10.1212/WNL.0000000000012753

**Published:** 2021-11-09

**Authors:** Steve Simpson-Yap, Edward De Brouwer, Tomas Kalincik, Nick Rijke, Jan A. Hillert, Clare Walton, Gilles Edan, Yves Moreau, Tim Spelman, Lotte Geys, Tina Parciak, Clement Gautrais, Nikola Lazovski, Ashkan Pirmani, Amin Ardeshirdavanai, Lars Forsberg, Anna Glaser, Robert McBurney, Hollie Schmidt, Arnfin B. Bergmann, Stefan Braune, Alexander Stahmann, Rodden Middleton, Amber Salter, Robert J. Fox, Anneke van der Walt, Helmut Butzkueven, Raed Alroughani, Serkan Ozakbas, Juan I. Rojas, Ingrid van der Mei, Nupur Nag, Rumen Ivanov, Guilherme Sciascia do Olival, Alice Estavo Dias, Melinda Magyari, Doralina Brum, Maria Fernanda Mendes, Ricardo N. Alonso, Richard S. Nicholas, Johana Bauer, Aníbal Sebastián Chertcoff, Anna Zabalza, Georgina Arrambide, Alexander Fidao, Giancarlo Comi, Liesbet Peeters

**Affiliations:** From the CORe (S.S.-Y., T.K.), Department of Medicine, and Neuroepidemiology Unit (S.S.-Y., N.N., A.F.), Melbourne School of Population & Global Health, University of Melbourne, Parkville, Australia; Menzies Institute for Medical Research (S.S.-Y.), University of Tasmania, Hobart, Australia; ESAT-STADIUS (E.D.B., Y.M., A.P.), KU Leuven, Belgium; Department of Neurology (T.K.), Melbourne MS Centre, Royal Melbourne Hospital, Parkville, Australia; MS International Federation (N.R., C.W.), London, UK; Department of Clinical Neuroscience (J.A.H., T.S., L.F., A.G.), Swedish MS Registry, Stockholm, Sweden; Department of Neurology (G.E.), CHU Pontchaillou, Rennes, France; Karolinska Institutet (T.S.), Solna, Sweden; Biomedical Research Institute–Data Science Institute (L.G., A.P., L.P.), Hasselt University, Belgium; Department of Medical Informatics (T.P.), University Medical Center Göttingen, Germany; Department of Computer Science and AI (C.G.), KU Leuven, Belgium; QMENTA (N.L.), Barcelona, Spain; Medpace Reference Laboratories (A.A.), Molecular Unit, Leuven, Belgium; iConquerMS People-Powered Research Network (R. McBurney, H.S.), Accelerated Cure Project for MS, Waltham, MA; NeuroTransData Study Group (A.B.B., S.B.), NeuroTransData, Neuburg, Germany; German MS-Register by the National MS Society (A. Stahmann), MS Forschungs- und Projektentwicklungs-gGmbH, Hannover, Germany; UK MS Register (R. Middleton, R.S.N.), Swansea University, UK; COViMS (A. Salter, R.J.F.), US; Division of Biostatistics (A. Salter), Washington University in St. Louis, St Louis, MO; Mellen Center for Multiple Sclerosis (R.J.F.), Cleveland Clinic, Cleveland, OH; Department of Neuroscience (A.v.d.W., H.B.), Central Clinical School, Monash University, Melbourne, Australia; Al-Amiri Hospital (R.A.), Kuwait City, Kuwait; Dokuz Eylul University (S.O.), Izmir, Turkey; Neurology Department (J.I.R.), Hospital Universitario de CEMIC; RELACOEM (J.I.R., R.N.A.), Buenos Aires, Argentina; Australian MS Longitudinal Study (I.v.d.M.), Menzies Institute for Medical Research, University of Tasmania, Hobart, Australia; Bulgarian SmartMS COVID-19 Dataset (R.I.), Sofia, Bulgaria; ABEM–Brazilian MS Patients Association (G.S.d.O., A.E.D.), São Paulo, Brazil; Danish Multiple Sclerosis Registry (M.M.), Department of Neurology, University Hospital Rigshospitalet, Glostrup, Denmark; Universidade Estadual Paulista (D.B.), Unesp, Faculdade de Medicina, Botucatu, Brazil; REDONE.br–Brazilian Registry of Multiple Sclerosis and Neuromyelitis Optica Spectrum Disorders (D.B., M.F.M.); Irmandade da Santa Casa de Misericórdia de São Paulo (M.F.M.), Brazil; Multiple Sclerosis University Center (R.N.A.), Ramos Mejia Hospital–EMA, Buenos Aires, Argentina; Imperial College London (R.S.N.); Swansea University (R.S.N.), Swansea, UK; Mental Health Area (J.B.); MS and Demyelinating Diseases (A.S.C.)., Hospital Británico de Buenos Aires, EMA, Argentina; Servei de Neurologia-Neuroimmunologia (A.Z., G.A.), Centre d’Esclerosi Múltiple de Catalunya, (Cemcat); Vall d’Hebron Institut de Recerca (A.Z., G.A.), Vall d’Hebron Hospital Universitari; Universitat Autònoma de Barcelona (A.Z., G.A.), Spain; and Institute of Experimental Neurology (G.C.), Ospedale San Raffaele, Milan, Italy.

## Abstract

**Background and Objectives:**

People with multiple sclerosis (MS) are a vulnerable group for severe coronavirus disease 2019 (COVID-19), particularly those taking immunosuppressive disease-modifying therapies (DMTs). We examined the characteristics of COVID-19 severity in an international sample of people with MS.

**Methods:**

Data from 12 data sources in 28 countries were aggregated (sources could include patients from 1–12 countries). Demographic (age, sex), clinical (MS phenotype, disability), and DMT (untreated, alemtuzumab, cladribine, dimethyl fumarate, glatiramer acetate, interferon, natalizumab, ocrelizumab, rituximab, siponimod, other DMTs) covariates were queried, along with COVID-19 severity outcomes, hospitalization, intensive care unit (ICU) admission, need for artificial ventilation, and death. Characteristics of outcomes were assessed in patients with suspected/confirmed COVID-19 using multilevel mixed-effects logistic regression adjusted for age, sex, MS phenotype, and Expanded Disability Status Scale (EDSS) score.

**Results:**

Six hundred fifty-seven (28.1%) with suspected and 1,683 (61.9%) with confirmed COVID-19 were analyzed. Among suspected plus confirmed and confirmed-only COVID-19, 20.9% and 26.9% were hospitalized, 5.4% and 7.2% were admitted to ICU, 4.1% and 5.4% required artificial ventilation, and 3.2% and 3.9% died. Older age, progressive MS phenotype, and higher disability were associated with worse COVID-19 outcomes. Compared to dimethyl fumarate, ocrelizumab and rituximab were associated with hospitalization (adjusted odds ratio [aOR] 1.56, 95% confidence interval [CI] 1.01–2.41; aOR 2.43, 95% CI 1.48–4.02) and ICU admission (aOR 2.30, 95% CI 0.98–5.39; aOR 3.93, 95% CI 1.56–9.89), although only rituximab was associated with higher risk of artificial ventilation (aOR 4.00, 95% CI 1.54–10.39). Compared to pooled other DMTs, ocrelizumab and rituximab were associated with hospitalization (aOR 1.75, 95% CI 1.29–2.38; aOR 2.76, 95% CI 1.87–4.07) and ICU admission (aOR 2.55, 95% CI 1.49–4.36; aOR 4.32, 95% CI 2.27–8.23), but only rituximab was associated with artificial ventilation (aOR 6.15, 95% CI 3.09–12.27). Compared to natalizumab, ocrelizumab and rituximab were associated with hospitalization (aOR 1.86, 95% CI 1.13–3.07; aOR 2.88, 95% CI 1.68–4.92) and ICU admission (aOR 2.13, 95% CI 0.85–5.35; aOR 3.23, 95% CI 1.17–8.91), but only rituximab was associated with ventilation (aOR 5.52, 95% CI 1.71–17.84). Associations persisted on restriction to confirmed COVID-19 cases. No associations were observed between DMTs and death. Stratification by age, MS phenotype, and EDSS score found no indications that DMT associations with COVID-19 severity reflected differential DMT allocation by underlying COVID-19 severity.

**Discussion:**

Using the largest cohort of people with MS and COVID-19 available, we demonstrated consistent associations of rituximab with increased risk of hospitalization, ICU admission, and need for artificial ventilation and of ocrelizumab with hospitalization and ICU admission. Despite the cross-sectional design of the study, the internal and external consistency of these results with prior studies suggests that rituximab/ocrelizumab use may be a risk factor for more severe COVID-19.

Disease-modifying therapies (DMTs) that act by immunomodulatory/immunosuppressive mechanisms are a mainstay of treatment of multiple sclerosis (MS) but can increase infection susceptibility.^1^ Cross-sectional^2^ and cohort^3-5^ studies suggest that comorbid conditions, age, sex, progressive MS phenotype, and higher disability increase risk for developing severe coronavirus disease 2019 (COVID-19).^3,5^ Some studies have also identified associations of certain DMT classes with COVID-19 severity.^3^ Using the French Covisep registry, the authors assessed 347 patients with MS with suspected/confirmed COVID-19, finding those treated with DMTs with a greater risk of systemic infection (alemtuzumab/cladribine/fingolimod/ocrelizumab/rituximab) had >4 times higher proportions with severe COVID-19 than DMTs with no infection risk (interferon beta/glatiramer acetate).^3^ Using the MuSC-19 retrospective cohort study, authors assessed COVID-19 severity among 844 patients with MS with suspected/confirmed COVID-19, finding that those treated with anti-CD20 DMTs (ocrelizumab/rituximab) had 2.4 times higher risk of severe COVID-19 compared to those treated with dimethyl fumarate.^5^ The COVID in MS (COViMS) Registry assessed characteristics of COVID-19 severity among 1,626 people with MS with suspected/confirmed COVID-19, finding that, compared to the untreated patients, ocrelizumab-treated (odds ratio [OR] 1.63) and rituximab-treated (OR 4.56) patients had higher frequencies of hospitalization, although no associations with intensive care unit (ICU) admission/ventilation or death were seen.^2^

Large and geographically inclusive cohorts are required to assess the risk of severe COVID-19 for specific DMTs. Accordingly, we established a global data-sharing initiative^6^ to investigate characteristics of COVID-19 severity in people with MS. We hypothesized that older age, progressive MS phenotype, and higher disability were associated with more severe COVID-19, while immunosuppressive DMTs (alemtuzumab/cladribine/fingolimod/ocrelizumab/rituximab) would be deleterious, but those with less infection risk (interferons/glatiramer acetate) would be associated with a less severe COVID-19.

## Methods

### Standard Protocol Approvals, Registrations, and Patient Consents

This study received approval from an ethics standards committee on human experimentation (institutional or regional) for any experiments using human participants (ethics committee of Hasselt University, CME2020/025). Other ethics information from data custodians includes the following.

MSBase data are provided with the consent of individual participants and principal investigators at each MSBase participating center. The German MS-Register was first approved by ethics committee of Julius-Maximilians-University of Würzburg (vote 142/12). After a switch was made to the web-based documentation system, further positive votes, for example, by the ethics committee of the Thuringia State chamber of physicians, followed by several ethics committees of different universities, were given, and all patients signed an informed consent.

Research participant protection was sought from the Washington University in St. Louis Institutional Review Board for housing COViMS Registry data, who determined it to be not human participants research and therefore exempt from active Institutional Review Board oversight at Washington University in St. Louis and did not require patient consent.

The patient data sent to analyses resulting in the Associations of DMT Therapies With COVID-19 Severity in Multiple Sclerosis study originated from a study approved by the ethics committee of the Faculdade de Medicina de Botucatu, Universidade Estadual Paulista under the internal review board number CAAE 31021220.2.0000.5411. All participants signed a written informed consent form before enrollment.

The Centre d’Esclerosi Múltiple de Catalunya cohort study was approved by the ethics committee of the Vall d'Hebron University Hospital (XMG-INT-2014-01), and all patients signed an informed consent.

Data from a core questionnaire on COVID-19 and relevant demographic/clinical information were reported by treating clinicians. The methods underlying the MS Data Alliance (MSDA) COVID-19 collaboration and measures thereof have been described previously.^6^ Briefly, treating clinicians entered information on a range of demographic, lifestyle, and MS-specific and COVID-19–specific clinical characteristics. Here, only age, sex, MS phenotype, disability, DMTs, smoking, body mass index (BMI), comorbid conditions, COVID-19 status, hospitalization, ICU admission, artificial ventilation, and death are described. Study participation was restricted to patients with MS who were ≥18 years of age; however, this article is limited to those with suspected/confirmed COVID-19.

Data were entered in 3 fashions: (1) direct entry to central platform; (2) patient-level data sharing via participating registries/cohorts, whereby MS registries and cohorts are regularly invited to share and upload their COVID-19 core dataset into the central data platform; and (3) aggregated data sharing via participating registries/cohorts, whereby some registries do not share data from individual patients but share aggregated results from specific queries.

Multidimensional contingency tables from 12 different data sources were merged, and then a combined anonymized dataset was reconstructed. Not all patients at each contributing data source necessarily participated in this study. Indeed, given the high proportion of patients with suspected and confirmed COVID-19, it is likely that a minority of patients at each center participated.

Data were entered for a given participant once, but information for that participant could be reentered, and these reentered data replaced the original record. This made for serial iterations of the analysis dataset, which were analyzed over time as the dataset expanded, thus allowing for assessment of temporal consistency of observed associations between the versions of the dataset. In this fashion, if associations were erratic in their appearance between iterations, this might suggest them to be statistical artifact, whereas consistency would indicate their veracity.

To improve the quality of the data continuously over time, we set up a data quality assessment and enhancement pipeline. This pipeline consists of 2 major parts: unambiguously defining new variables that are used in downstream analysis (e.g., defining COVID-19 suspected and confirmed cases, categorizing continuous variables to allow aggregation of the counts) and predefining pass/fail criteria for variables (e.g., negative ages, unrealistically high numbers for height). Variables that fail were flagged, and registry custodians were contacted to repair failed variables in the next upload. Simultaneously, failed variables were cleaned and preprocessed so that records could be incorporated into the downstream analysis.

### Variables

Definitions for all terms were provided to data partners and were available on the MSDA platform. Clinicians made all judgments regardless of how data were entered. This data dictionary is available by contacting the corresponding author.

COVID-19 status was defined as confirmed, based on a positive diagnostic test, or suspected, based on clinician judgment.

Hospitalization was queried as admission to hospital because of COVID-19 (suspicious) infection. ICU admission was queried as stay in ICU because of COVID-19 (suspicious) infection. Requiring artificial ventilation was queried as ventilation needed during hospital stay. Death due to COVID-19 was queried as “did the patient die because of the (suspected) COVID-19 infection?”

Patient age was categorized into 3 age groups: 18 to 49, 50 to 69, and ≥70 years. MS phenotype was grouped into relapsing-remitting MS (RRMS) and progressive MS (secondary and primary progressive MS).

Disability was assessed by the Expanded Disability Status Scale (EDSS)^7^ or Neurostatus.^8^ Disability was dichotomized into scores of 0 to 6.0 and >6.0. Comorbid conditions were queried, including cardiovascular disease, hypertension, diabetes, chronic liver disease, kidney disease, other neurologic/neuromuscular disorder, lung disease, or malignant neoplasia. BMI was categorized as nonobese (BMI ≤30 kg/m^2^) and obese (BMI >30 kg/m^2^). Current smoker status was queried as yes or no. Current DMT use was queried, including alemtuzumab, cladribine, dimethylfumarate, fingolimod, glatiramer acetate, interferons, natalizumab, ocrelizumab, rituximab, siponimod, teriflunomide, or other DMT, which was queried as on another drug not listed. Due to patient numbers <20 among the suspected/confirmed COVID-19 cases, siponimod (n = 12) was aggregated with other DMT. Note that aggregation of siponimod with fingolimod did not materially change results (data not shown).

### Statistical Analysis

Associations with hospitalization, ICU admission, ventilation, and death were assessed with multilevel mixed-effects logistic regression, random effects grouped by data source, as univariable and adjusted for age, sex, MS phenotype, and disability. Multilevel mixed-effects logistic regression is an appropriate methodology wherein multiple data sources with heterogeneity in cohort characteristics or methods are included. This method applies fixed and random effects to the model, accounting for intraclass correlation for observations within data sources, providing aggregate statistics for the measures of association of the independent model covariates. While all data sources used the same data entry framework and core questionnaire, there are differences in the modes of clinical practice, for MS- and infection-related care, as well as clinical and demographic characteristics, which need be accounted for and for which this statistical method is suited.

Subgroup analyses were also undertaken whereby data on comorbid condition, BMI, and smoking were available, allowing additional adjustment for these covariates. All analyses were complete case.

For DMTs, individual DMTs were first compared with dimethyl fumarate. Despite leading to lymphopenia in some patients, dimethyl fumarate has not been associated with increased infection risk,^9^ and its biological mechanism of action is unlikely to interfere with anti–severe acute respiratory syndrome coronavirus 2 (SARS-CoV-2) immunologic response^10^ while being common in the sample. Of note, interferons and glatiramer acetate were considered as potential comparators, but the lack of failures across all 4 outcomes for these DMTs precluded their being the reference group. Next, ocrelizumab, rituximab, and untreated were compared against all other pooled DMTs. Finally, ocrelizumab and rituximab were evaluated vs natalizumab to assess ascertainment bias because natalizumab-treated patients present for infusions every 28 to 42 days compared to biannual infusions for anti-CD20 DMTs.

Adjustment for multiple comparisons was undertaken with the family-wise Holm step-down method, such that within each hypothesis and within models 1 and 2, statistical tests were ranked by lowest *p* value and significance threshold evaluated relative to the number of statistical tests within that family. Associations reaching significance after this adjustment are annotated as such in tables.

Data analyses were carried out with STATA/SE 16.0 (StataCorp, College Station, TX).

### Data Availability

Persons interested in acquiring the anonymized data underlying this analysis can inquire with a senior author (L.P.) to make requests. In addition, supplementary tables not included in the main body text can be found in the preprint version of the article, accessible at doi.org/10.1101/2021.02.08.21251316.

## Results

The cohort comprised 2,340 patients, of whom 657 (28.1%) had suspected COVID-19 and 1,683 (71.9%) had confirmed COVID-19. Among suspected/confirmed COVID-19 cases, which made up the primary analysis dataset, 20.9% were hospitalized, 5.4% were admitted to ICU, 4.1% required artificial ventilation, and 3.2% died. Proportions were slightly higher among confirmed COVID-19 cases (Table 1).

**Table 1:**
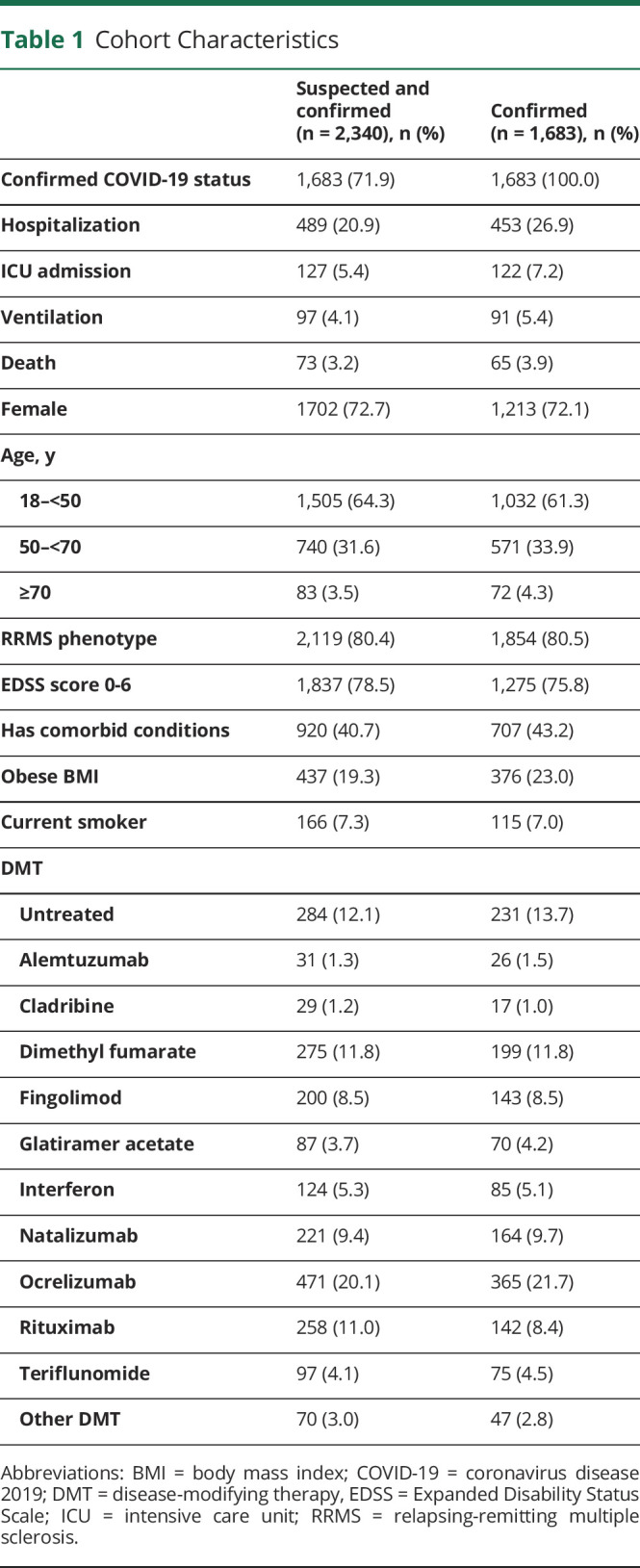
Cohort Characteristics

### Cohort Characteristics

Data sources were located in (1) Sweden (n = 290); (2) Australia, Belgium, Brazil, Kuwait, Romania, Saudi Arabia, and Turkey (n = 97); (3) Argentina, Chile, Colombia, Ecuador, Honduras, and Mexico (n = 159); (4) Bulgaria (n = 3); (5) Germany and Italy (n = 45); (6) Denmark (n = 56); (7) Brazil (n = 96); (8) Australia, Bahamas, Belgium, Czech Republic, Finland, France, the Netherlands, New Zealand, Serbia, Spain, the United Kingdom, and the United States (n = 114); (9) Germany (n = 41); (10) the United States and Canada (n = 1,161); (11) the United Kingdom (n = 131); and (12) Spain (n = 147). Sources 2 and 10 had higher proportions with confirmed COVID-19, and sources 4 and 5 had higher proportions with nonsuspected COVID-19. Among suspected/confirmed COVID-19 cases, hospitalization was higher in source 11 and lower in sources 2, 4, 5, and 7; ICU admission was higher in sources 3, 9, and 10 and lower in sources 2, 4, 7, 9, 11, and 12; ventilation was higher in sources 3 and 8 and lower in sources 2, 4 through 7, 9, and 11; and death was higher in source 11 and lower in sources 2 and 4 through 9. Results were comparable on restriction to confirmed-only COVID-19 (data not shown).

Compared to dimethyl fumarate (76.1%), lower proportions of female patients were untreated (70.7%) or treated with interferon (69.4%) or ocrelizumab (67.5%, eTable 1, links.lww.com/WNL/B520). Larger proportions of those 50 to 69 and ≥70 years of age were untreated (42.8%, 13.4%) or treated with ocrelizumab (34.2%, 1.5%), teriflunomide (49.5%, 3.1%), or other DMTs (37.7%, 5.8%) than with dimethyl fumarate (29.9%, 0.8%). Greater proportions of patients with progressive MS were untreated (44.9%) or treated with ocrelizumab (20.2%), rituximab (21.8%), or other DMTs (31.9%) than with dimethyl fumarate (8.0%). Of patients with greater disability (EDSS score >6), higher proportions were either untreated (35.9%) or treated with ocrelizumab (27.0%) or other DMTs (34.8%) than with dimethyl fumarate (7.6%). Similar results were seen among confirmed-only COVID-19 (data not shown).

**Figure 1:**
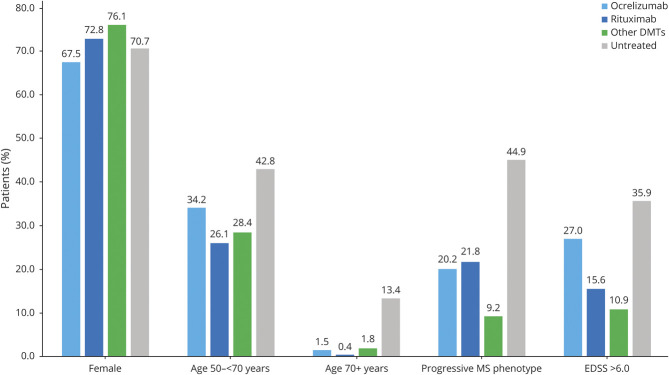
Characteristics of Patients Treated With Ocrelizumab, Rituximab, or Other DMTs or Untreated, by Sex, Age, MS Phenotype, and Disability DMT = disease-modifying therapy; EDSS = Expanded Disability Status Scale; MS = multiple sclerosis.

In evaluations of ocrelizumab and rituximab compared to pooled other DMTs and the untreated (Figure 1), anti-CD20 DMTs were comparable by sex and MS phenotype, but a greater proportion of ocrelizumab-treated patients were 50 to 69 years of age and had an EDSS score ≤6. Greater proportions of anti-CD20–treated patients were of the progressive MS phenotype or had an EDSS score >6 than those treated with the pooled other DMTs, while the untreated were typically older, had progressive MS phenotype, and had higher disability.

### COVID-19 Severity by Clinical/Demographic Characteristics

Demographic and clinical characteristics of hospitalization and ICU admission (eTable 1, links.lww.com/WNL/B520) and need for artificial ventilation and death (eTable 2, links.lww.com/WNL/B521) among suspected/confirmed COVID-19 cases were assessed. In multivariable models, male sex was associated with 61% and 92% greater risks of hospitalization and death, while older age was positively associated with hospitalization and death. Progressive MS phenotype was associated with 68% higher risk of hospitalization. Higher EDSS score was associated with higher risks of all outcomes, including 279% higher hospitalization, 211% higher ICU admission, 281% higher ventilation, and 893% higher death frequencies. Among confirmed-only COVID-19, all associations persisted except for age >70 years, which became nonsignificant (data not shown).

In the subset of data sources with data (comorbid conditions 84.2%, BMI 55.6%, smoking 79.3%), having one of the specified comorbid conditions queried showed a positive trend with increased risk of death (adjusted OR [aOR] 2.91), while obese BMI had a 152% higher risk of hospitalization, 312% higher risk of ICU admission, and 445% higher risk of requiring ventilation. Smoking was not associated with any outcomes.

Adjustment for multiple comparisons found most associations of sex, age, MS phenotype, and EDSS score with COVID-19 outcomes persisted, as did BMI and comorbid conditions.

### COVID-19 Severity by DMT

Compared to dimethyl fumarate, rituximab use was associated with greater risks of hospitalization (aOR 2.43, 95% confidence interval [CI] 1.48, 4.02), ICU admission (aOR 3.93, 95% CI 1.56, 9.89), and artificial ventilation (aOR 4.00, 95% CI 1.54, 10.39, Table 2). Ocrelizumab showed similar associations for hospitalization (aOR 1.56, 95% CI 1.01, 2.41) and ICU admission (aOR 2.30, 95% CI 0.98, 5.39) but not artificial ventilation (aOR 1.04, 95% CI 0.41, 2.64).

**Table 2:**
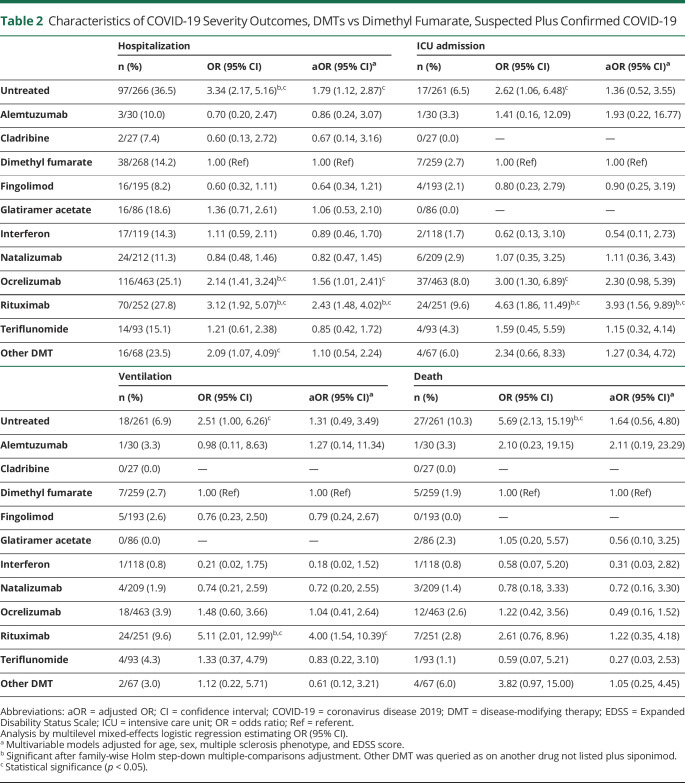
Characteristics of COVID-19 Severity Outcomes, DMTs vs Dimethyl Fumarate, Suspected Plus Confirmed COVID-19

**Figure 2:**
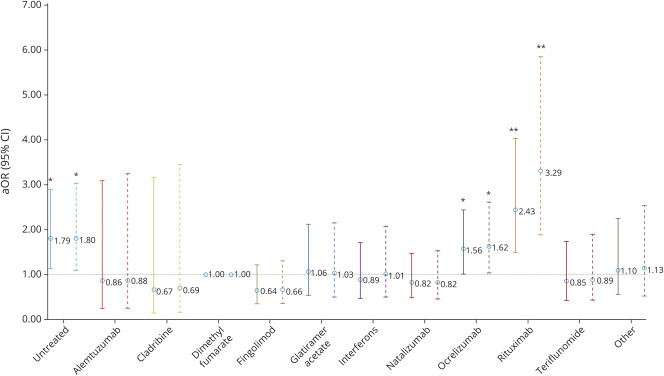
Risk of Hospitalization by DMT Among Suspected/Confirmed (Solid Line) and Confirmed-Only (Dashed Line) Cases Disease-modifying therapies (DMTs) compared to dimethyl fumarate adjusted for age, sex, multiple sclerosis phenotype, and Expanded Disability Status Scale score. Other DMTs also include siponimod. aOR = adjusted odds ratio; CI = confidence interval. *p < 0.05, **p < 0.001.

**Figure 3:**
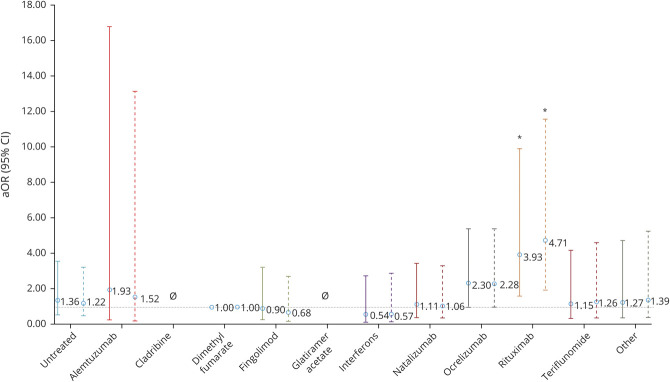
Risk of ICU Admission by DMT Among Suspected/Confirmed (Solid Line) and Confirmed-Only (Dashed Line) Cases Disease-modifying therapies (DMTs) were compared to dimethyl fumarate, adjusted for age, sex, multiple sclerosis phenotype, and Expanded Disability Status Scale score. Other DMTs also include siponimod. Null set denotes analyses that could not be undertaken due to no events occurring in the exposed group. aOR = adjusted odds ratio; CI = confidence interval. *p < 0.05.

**Figure 4:**
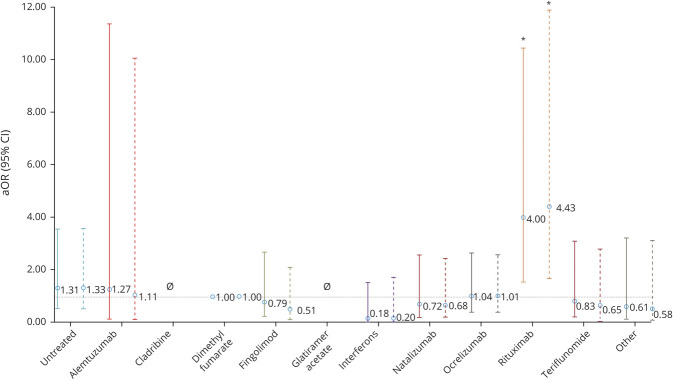
Risk of Artificial Ventilation by DMT Among Suspected/Confirmed (Solid Line) and Confirmed-Only (Dashed Line) Cases Disease-modifying therapies (DMTs) were compared to dimethyl fumarate adjusted for age, sex, multiple sclerosis phenotype, and Expanded Disability Status Scale score. Other DMTs also includes siponimod. Null set denotes analyses that could not be undertaken due to no events occurring in the exposed group. aOR = adjusted odds ratio; CI = confidence interval. *p < 0.05.

No DMTs were associated with death. Untreated patients had increased risk of hospitalization (aOR 1.79, 95% CI 1.12, 2.87), but no independent associations with other outcomes were seen. These associations persisted among confirmed-only COVID-19 (Figures 2–4). Moreover, even on comparison to DMTs other than dimethyl fumarate as a post hoc analysis, rituximab consistently showed stronger associations with outcomes than ocrelizumab for hospitalization and ICU admission and was solely associated with requiring artificial ventilation (data not shown).

On adjustment for multiple comparisons, among suspected plus confirmed cases, rituximab associations with hospitalization and ICU admission remained significant, although that for ventilation did not. However, among confirmed-only cases, the rituximab associations with hospitalization, ICU admission, and need for artificial ventilation remained significant after multiple-comparisons adjustment. The association of ocrelizumab with increased hospitalization did not remain significant in any analysis.

### COVID-19 Severity: Anti-CD20 DMTs vs Pooled Other DMTs

Compared to all other DMTs, those using rituximab had higher risks of hospitalization (aOR 2.76, 95% CI 1.87, 4.07, Table 3), ICU admission (aOR 4.32, 95% CI 2.27, 8.23), and artificial ventilation (aOR 6.15, 95% CI 3.09, 12.27). Ocrelizumab showed similar trends for hospitalization (aOR 1.75, 95% CI 1.29, 2.38) and ICU admission (aOR 2.55, 95% CI 1.49, 4.36) but not ventilation (aOR 1.60, 95% CI 0.82–3.14). Neither rituximab (aOR 1.72, 95% CI 0.58, 5.10) or ocrelizumab (aOR = 0.73, 95% CI = 0.32, 1.70) was associated with risk of death. Untreated patients had increased risks of hospitalization (aOR 2.05, 95% CI 1.43, 2.94), ventilation (aOR 2.07, 95% CI 1.01.4.22), and death (aOR 2.53, 95% CI 1.24.5.15). These results persisted among confirmed-only COVID-19 cases (data not shown).

**Table 3:**
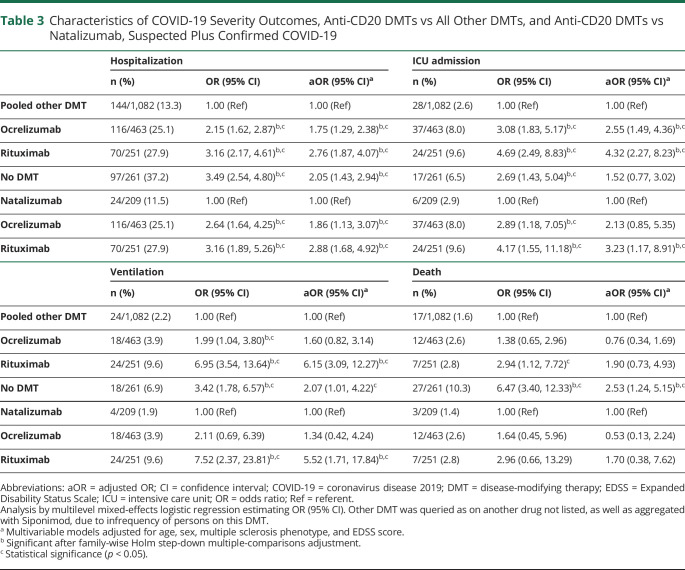
Characteristics of COVID-19 Severity Outcomes, Anti-CD20 DMTs vs All Other DMTs, and Anti-CD20 DMTs vs Natalizumab, Suspected Plus Confirmed COVID-19

After adjustment for multiple comparisons, all rituximab and ocrelizumab associations persisted, while that for the untreated patients persisted for hospitalization and death. Among confirmed-only cases, rituximab associations remained significant after multiple-comparisons adjustment, while that for ocrelizumab persisted only for hospitalization and for untreated for hospitalization and ventilation.

### COVID-19 Severity, Anti-CD20 DMTs vs Natalizumab

Compared to natalizumab, rituximab was associated with higher risks of hospitalization (aOR 2.88, 95% CI 1.68.4.92), ICU admission (aOR 3.23, 95% CI 1.17.8.91), and ventilation (aOR 5.52, 95% CI 1.71.17.84). Ocrelizumab showed similar trends for hospitalization (aOR 1.86, 95% CI 1.13.3.07) but did not reach significance for ICU admission and was not associated with ventilation. Neither rituximab (aOR 1.34, 95% CI 0.27.6.56) nor ocrelizumab (aOR 0.48, 95% CI 0.11.2.07) was associated with increased risk of death. Results were similar among confirmed-only COVID-19 (data not shown).

On adjustment for multiple comparisons, rituximab associations with hospitalization, ICU admission, and need for artificial ventilation all persisted, while that for ocrelizumab persisted only for hospitalization. On restriction to confirmed-only cases, this was also seen.

### Stratification Sensitivity Analyses

To assess whether the associations seen for ocrelizumab and rituximab were genuinely a function of the DMTs rather than the characteristics of patients commonly treated with these medications (older, progressive MS, higher disability), we undertook stratified analyses evaluating associations between DMTs and hospitalization, ICU admission, need for artificial ventilation, and death among persons >70 vs ≤70 years of age, among those with RRMS vs progressive, and among patients with EDSS scores ≤6 vs >6. By age, ocrelizumab and rituximab associations with outcomes were seen only among those ≤70 years of age. By MS phenotype, ocrelizumab and rituximab consistently showed that associations with outcomes were roughly 2 to 3 times stronger in magnitude among those with the RRMS phenotype. By EDSS score, ocrelizumab and rituximab associations with hospitalization were roughly comparable, but associations with ICU admission and ventilation were 2 to 3 times stronger in magnitude among those of EDSS score ≤6. These results indicate that the observed associations were a function of the DMT, not the underlying risk profile (data not shown).

## Discussion

In the largest sample of people with MS with suspected and confirmed COVID-19 to date, we demonstrated that the anti-CD20 DMTs rituximab and ocrelizumab were associated with more severe COVID-19. Compared to dimethyl fumarate, pooled other DMTs, and natalizumab, anti-CD20 DMTs were associated with higher risks of hospitalization and ICU admission, while only rituximab was associated with greater risk of requiring artificial ventilation. Comparison to natalizumab is particularly important, showing that anti-CD20 associations do not likely reflect ascertainment bias. Regardless of comparator, rituximab consistently showed stronger associations with outcomes than ocrelizumab for hospitalization and ICU admission and was solely associated with requiring artificial ventilation. It is important to note that this was done as a post hoc exploration, not an a priori plan, given the specific hypotheses of the study. At the same time, untreated patients had a significantly greater risk of death compared to persons treated with dimethyl fumarate and pooled non–anti-CD20 DMTs, although this attenuated markedly on adjustment. Moreover, DMT associations were not merely driven by older age, progressive MS phenotype, or higher disability. We also found that older age, progressive MS phenotype, and higher disability were overrepresented among patients with MS with more severe COVID-19. In subanalyses in which data were available, DMT associations were robust to further adjustment for comorbid conditions, BMI, and smoking status. Results were generally consistent on adjustment for multiple comparisons.

An increasing array of highly effective DMTs with heterogeneous modes of effect have become available for people with MS but can increase infection risk.^1,11^ This has raised concern during the COVID-19 pandemic, and national studies have investigated risk factors for severe COVID-19 disease in people with MS. In the French Covisep study,^3^ of 347 patients with MS (42.1% confirmed COVID-19), older age, progressive MS phenotype, higher disability, and comorbid conditions were associated with COVID-19 severity. The authors’ pooled DMTs with moderate/high risk of systemic infection (fingolimod/ocrelizumab/rituximab/cladribine/alemtuzumab) were associated with 4.2 times higher COVID-19 severity score than DMTs with no systemic infection risk (interferon beta/glatiramer acetate). This amalgamation of DMTs is a limitation because, while comparable in terms of their infection risk, these DMTs have markedly different modes of action, especially in relation to the immunologic response to SARS-CoV-2.^10^ More recently, the Italian MuSC-19 national registry study of 593 suspected and 191 confirmed COVID-19 cases found that anti-CD20 DMT use was associated with 2.6 times greater risk of severe COVID-19 compared to dimethyl fumarate use, adjusted for region, age, sex, MS phenotype, and recent methylprednisolone use.^5^ Finally, the COViMS study found that, compared to untreated patients, rituximab- and ocrelizumab-treated patients had 4.6 and 1.6 times greater odds of hospitalization, although no significant associations were seen for other outcomes.^2^ This disparity with our and previous results may reflect their use of the untreated as the comparator, as well as differences in the multivariable model covariates. It should be acknowledged that the COViMS sample comprised a meaningful proportion of our total sample (n = 1,161 of 2,340, 49.6%), and while the total cohort analyzed and analysis methods used here differ, results for this cohort have been described previously. The remainder of the cohort described here has not been previously described.

Indeed, the issue of comparator is a point of particular attention in our study; we compared individual DMTs to dimethyl fumarate, and then, because they showed significant associations here, the anti-CD20 DMTs were compared against all other DMTs, and finally these were compared to natalizumab. Dimethyl fumarate was identified as a suitable comparator, being common in the sample and also used for the MuSC-19 study.^5^ The untreated were not regarded as an appropriate primary comparator because these patients differed markedly from the rest of the cohort in age, disability, and MS phenotype. This latter comparator is of particular importance because it assesses ascertainment bias as a potential explanation for the associations seen for anti-CD20 DMTs. Both the anti-CD20 DMTs and natalizumab require patients to come in for DMT infusion at 6- and 3-month intervals, respectively. Thus, if the anti-CD20 DMT associations with COVID-19 severity were merely a function of more regular hospital attendance and thus potential for COVID-19 symptoms to be identified and treatment initiated, then there should be no difference from natalizumab. In fact, we found that the associations of ocrelizumab and rituximab with hospitalization and ICU admission and rituximab with need for artificial ventilation persist.

Untreated patients showed consistent positive trends toward associations with hospitalization, ICU admission, and requiring ventilation, albeit attenuating on adjustment for age, sex, MS phenotype, and disability. This is in keeping with prior results. Louapre et al.^3^ found higher frequencies of severe COVID-19 among the untreated vs treated (46.0% vs 15.5%), although this difference did not persist on adjustment. Sormani et al.^5^ compared untreated to dimethyl fumarate-treated patients, finding that they were 2.83 times more likely to have severe COVID-19, although this disappeared on adjustment (aOR 1.04). The lack of independence of the untreated associations here and previously likely reflects the untreated comprising, to variable degrees, people with a more benign MS course or other reasons not to use DMTs, so adjustment for MS phenotype and disability largely captures differences in COVID-19 severity. That said, it is important to acknowledge that the heterogeneity of this patient population may extend beyond differences in clinical phenotype and likely includes a range of patient and region-level idiosyncrasies for which we were unable to account.

The directions of effect in the associations of rituximab and ocrelizumab were consistent for the hospitalization and ICU admission outcomes, although those for rituximab were stronger and only rituximab showed an association with requiring artificial ventilation. It is possible that differences in biology, due to differences in provenance or affinity for the CD20 protein^12,13^ or differences in mechanisms of cytotoxicity,^14^ may underlie some of this difference. More likely, these differences represent unmeasured confounding because the dataset, while large, was limited in the number of characteristics assessed, so potentially relevant factors like socioeconomic status and access to care or factors affecting respiratory health could not be assessed. In addition, parameters such as time on treatment or serum immunoglobulin load, which would have been valuable to explore for explanations for differences in associations between these 2 DMTs, were not available. That said, there was general internal consistency, and our results are broadly in line with those seen in other studies,^3,5^ providing external consistency. This preliminary observation is worth exploring in laboratory studies.

That anti-CD20 DMTs were not associated with death conflicts with the results seen for the other outcomes, as well as with the MuSC-19 study, which found a positive trend between anti-CD20 DMTs and death. The issue may lie in ascertainment bias, with fewer of the older patients included in our sample: we had only 9.1% of confirmed COVID-19 cases >60 years of age vs 17.7% in the MuSC-19 cohort.^5^ It is also possible that the infrequency of deaths in our cohort (n = 73, 3.2%) may have limited our statistical power to assess this relationship. That said, our number of deaths was actually greater than in previous studies (n = 12–54), and we did demonstrate similar deleterious associations of older age and higher disability with death seen previously.^2,3,5^ The potential impacts of these DMTs on death caused by COVID-19 should be further explored.

In contrast to prior clinic-based studies, our cohort focused on a predefined limited set of demographic and clinical characteristics.^6^ Thus, we could not assess other clinical features, particularly prior MS clinical course and DMT use, paraclinical information such as radiologic burden of MS, or the nuanced details of COVID-19 onset and evolution. Another limitation of our data is that they likely comprise greater proportions of severe cases requiring medical attention. One particular element lacking in our data is treatment duration or duration since treatment; both may have bearing on the degree of B-cell depletion and thus on COVID-19 severity. This information was included in the core questionnaire, but the level of missingness was too high to be a component of analyses. These data would have been useful in better describing and explaining the differences in the associations between rituximab and ocrelizumab, but unfortunately, this information was not available here. In addition, data on steroid use and other DMTs that might have affected clinical progression or resulted in less severe COVID-19 were not available.

Heterogeneity in the definitions of exposure and outcomes and in patient inclusion among the data sources is a known problem in combining multiple data sources. Related to this are the differences in protocols for hospital and ICU admission and initiation of artificial ventilation between hospitals, as well as differences in the availability/use of DMTs between countries.^15^ To ensure that our results were not being driven by single influential data sources, we undertook all analyses using random-effects logistic regression, as well as serial-exclusion sensitivity analyses. These analyses showed that, while there was some variation in the magnitudes and significance of associations, trends tracked as seen for the whole cohort, indicating that the results were not driven by a specific data source.

Another issue lies in the anonymous nature of the data entry, such that patients may be entered more than once in different data sources. We are unable to account for whether participants already participated or had their data entered in another study because there is no identifying information to assess this or any query of prior participation in the survey.

Another issue is the nature of the data aggregation, with some data sources providing individual patient-level data but others only tabulations of discrete categorical terms. Thus, we were obliged to use 3-level categories of age, 2-level EDSS scores, and 2-level BMIs rather than more exact values of each or 2-level MS phenotype (RRMS/progressive) rather than individual MS phenotype (RRMS/secondary progressive MS/primary progressive MS). That said, these levels are generally aligned with the levels of each associated with increased COVID-19 severity.

This data aggregation process and resultant limitation of the number of dimensions of model covariates thus required a somewhat simplified definition of underlying clinical severity as used in the stratified analyses by age, MS phenotype, and disability. We are also limited in not having additional clinical detail such as relapse activity or MRI parameters, which would have allowed greater depth of assessment of underlying disease risk phenotype. That said, older age, progressive MS phenotype, and higher disability have been consistently demonstrated as risk factors for COVID-19 severity across our results and previous studies,^2,3,5^ so if the DMT associations were merely a function of underlying clinical susceptibility, it should be expected that analyses stratified by these covariates would show some signal. That they did not is indicative of a true association.

The information collected through the different sources does not provide us with detailed information about validation of the COVID-19 diagnosis. Whether patients remained in the suspected group because of discrepancy between clinical and laboratory assessment or other reasons is unknown. Because of these diagnostic uncertainties, we opted to perform 2 analyses, 1 among group with suspected and confirmed COVID-19 and 1 in confirmed COVID-19 only.

Another potential issue is the representativeness of the included patients from each of the noncentral data sources. While there were no inclusion or exclusion criteria beyond the patients needing to have MS and be at least 18 years of age, the clinicians entering data for their patients would likely bias to enter data on patients who had suspected or confirmed COVID-19; this was reflected in the low proportion of the sample without suspected/confirmed COVID-19 (data not shown). However, such bias is typical of all the clinic-based studies of this sort. Our cohort characteristics are typical of people with MS, including female, RRMS phenotype, and low disability preponderance, so the extent to which this generalizability may affect the analyses here is likely minimal.

The fact that inverse trends were seen for smoking status and COVID-19 is puzzling and not in line with expectations of a deleterious association with a respiratory condition. The frequency of current smokers in the cohort is low compared to other cohorts,^16-18^ which may account for the absence of associations. In addition, smoking status was not available from all data sources, so a material proportion (20.7%) of the sample had smoking status missing. The Covisep study^3^ also found an inverse trend with COVID-19 severity, and the COViMS study^2^ found no associations. The explanation for this absence of a deleterious impact of smoking on COVID-19 severity may bear exploration.

In the largest population yet studied, we have shown that patients with MS treated with the anti-CD20 DMTs rituximab and ocrelizumab are at higher risk of more severe COVID-19 compared to those treated with dimethyl fumarate, pooled other DMTs, and natalizumab. This risk is additional to the risk associated with demographic and clinical characteristics, with older age, progressive MS phenotype, and higher disability all showing deleterious relationships with COVID-19 severity. These results agree with smaller cohort studies and suggest that the risk vs benefit of continued or new exposure to CD20-depleting treatment strategies compared to other DMTs needs to be considered in the context of the ongoing COVID-19 pandemic.

## Publication History

This manuscript was prepublished in MedRXiv: doi.org/10.1101/2021.02.08.21251316. Received by Neurology February 12, 2021. Accepted in final form August 27, 2021.

## Glossary

aOR: adjusted OR
BMI: body mass index
CI: confidence interval
COVID-19: coronavirus disease 2019
DMT: disease-modifying therapy
EDSS: Expanded Disability Status Scale
ICU: intensive care unit
MS: multiple sclerosis
MSDA: MS Data Alliance
OR: odds ratio
RRMS: relapsing-remitting MS
SARS-CoV-2: severe acute respiratory syndrome coronavirus 2
